# Techno-stress and teaching innovation among university teachers in the intelligent era: a path analysis of job burnout and AI self-efficacy

**DOI:** 10.3389/fpsyg.2026.1861301

**Published:** 2026-08-05

**Authors:** Qiong Wu, Xuelin Yang, Minghuan Tang

**Affiliations:** 1 School of Industrial Education and Technology, King Mongkut’s Institute of Technology Ladkrabang, Bangkok, Thailand; 2 School of Physical Education, Beibu Gulf University, Qinzhou, Guangxi, China

**Keywords:** AI self-efficacy, job burnout, teaching innovation, techno-stress, university teachers

## Abstract

**Background:**

In the context of deep integration of intelligent technologies into educational practice, how to balance the pressure from technology application with the intrinsic demands of teaching innovation, has become an important issue. Job burnout reflects an individual’s state of emotional exhaustion under long-term techno-stress, and AI self-efficacy indicates an individual’s confidence in their ability to use AI tools. There is still a lack of a clear explanation for how the two are jointly related to the relationship between techno-stress and teaching innovation. Based on the Job Demands-Resources Model, this study constructed a chain mediation model aimed at systematically examining the relationships among the variables.

**Method:**

The study adopted a stratified random sampling method to collect valid questionnaire data from a total of 819 university teachers, and conducted model checking and path effect analysis based on CB-SEM.

**Results:**

The results showed that techno-stress had a significant negative correlation with teaching innovation. The results of the mediation analysis indicate that the mediation effects of job burnout and AI self-efficacy account for 33.60% and 20.00% of the total effect, respectively, while the chained mediation effect resulting from both factors accounts for 14.13% of the total effect. Significant differences in teaching innovation levels were found across teacher groups divided by gender and age.

**Conclusion:**

The research results clarify the synergistic mechanism of external organizational factors and internal psychological factors driving teaching innovation, and have important implications for promoting the continuous teaching innovation of university teachers in the intelligent era.

## 1 Introduction

In the intelligent era, the rapid development of new technologies such as artificial intelligence (AI) is profoundly reshaping the teaching environment and disciplinary knowledge systems, while also enabling the fulfillment of students’ individualized and varied educational requirements. However, these changes collectively constitute unavoidable practical challenges in the teaching practices of university teachers (Sjöberg and Bergdahl, 2025). Teaching innovation, defined as a deliberate process of instructional improvement in which teachers introduce novel concepts, procedures, tools, or strategies to stimulate student engagement, enhance learning outcomes, and achieve educational goals (Huang et al., 2025). It has become a key pathway for university teachers to address practical challenges and achieve educational objectives in complex educational contexts amid the rapid evolution of the technology-driven educational ecosystem (Antonio et al., 2024). Against this backdrop, the results of a survey and research focusing on teaching innovation in higher education show that the adoption of data-driven solutions integrated with AI can achieve an outstanding course pass rate of over 93% and a knowledge retention rate of 74.8%, significantly enhancing teaching efficiency (Chen Z. et al., 2026). Thus, the deep integration of AI technology with teaching innovation is not only an effective means to enhance teaching outcomes, but also an inevitable choice for university teachers to adapt to contemporary changes and respond to practical challenges.

AI holds great potential for empowering teaching and curriculum innovation and supporting the sustainable development of this field, yet the complexity of its practical application cannot be overlooked (Su et al., 2026). The deep integration of AI technology and teaching practice has given rise to techno-stress among the teaching staff. This pressure stems from the ever-increasing demands for digital literacy and the uncertainty surrounding technological development, leaving teachers feeling anxious and helpless, which in turn hinders the advancement of teaching innovation (Hernández, 2025). Under such circumstances, teachers are prone to develop job burnout, a state that continuously consumes their psychological resources and innovative motivation, alongside a diminished intrinsic willingness toward experimenting with novel teaching strategies (Caracuel Cáliz et al., 2025). In contrast to the above-mentioned negative factors, the AI self-efficacy (AISE) has stimulated the vitality of teachers in exploring and practicing AI tools, injecting positive psychological momentum into teaching innovation (Al-Mahdy and Elwakil, 2026). Although existing research has separately examined the pairwise relationships among techno-stress, job burnout, AISE, and teaching innovation, the mechanisms through which job burnout and AISE function in the relationship between techno-stress and teaching innovation require further clarification. In particular, the potential chain of effects between the two still requires systematic empirical verification and theoretical interpretation among university teachers. To this end, this study attempts to construct a chain mediation model. By revealing the psychological process behind this relationship, it aims to provide a new theoretical perspective and practical entry point for maintaining the psychological resilience of teachers and stimulating the vitality of teaching innovation in the intelligent era.

### 1.1 Theoretical basis

The Job Demands-Resources Model (JD-R Model) (Demerouti et al., 2001) states that an individual’s psychological state and behavioral performance in the work environment depend on the dynamic balance between job demands and job resources. This model holds that excessively high job demands can lead to the depletion of an individual’s resources through the health impairment path, while abundant job resources can stimulate an individual’s active behavior through the motivational gain path. Moreover, there is an interaction between the two paths: excessive consumption of job demands weakens an individual’s ability to accumulate and mobilize job resources, thereby inhibiting the activation of the motivational path (Bakker and Demerouti, 2017). For teachers in the intelligent era, techno-stress, as a new job requirement, stems from the rapid iteration of digital teaching tools and AI systems and their deep integration into teaching scenarios. When teachers are forced to continuously respond to technological updates or meet requirements such as digital teaching evaluations, their psychological and emotional resources become persistently depleted. Under these ongoing pressures, job burnout tends to become more severe (Tu et al., 2025). This state of exhaustion is closely related to the richness of an individual’s inner psychological resources. When teachers experience burnout, their psychological resources are already depleted. This scarcity of resources makes it difficult for them to develop a sense of mastery and confidence in new technologies, thereby undermining their self-efficacy (Testa et al., 2025). In contrast, high-AISE teachers commonly view AI as both a driver of instructional innovation and an asset for administrative productivity (Hastomo et al., 2025). This connection from external job requirements to internal resource states theoretically echoes the core viewpoint of JD-R Model: job requirements and job resources are interrelated and jointly determine an individual’s degree of psychological exhaustion and tendency toward active behavior. Based on JD-R Model, this study aims to explore the underlying relationships among techno-stress, job burnout, AISE, and teaching innovation among university teachers.

### 1.2 Techno-stress and teaching innovation

Techno-stress refers to a negative psychological state that individuals experience when they cannot effectively cope with technology-related demands, often manifested as anxiety, fatigue, and reduced self-efficacy (Kumar, 2024). Research has found that techno-stress is significantly negatively correlated with teaching innovation (Hernández, 2025). For example, the study by Wu et al. (2022) confirmed that techno-stress significantly hinders teachers’ innovative behaviors in integrating technology into teaching. The psychological tension and emotional burden brought about by techno-stress can weaken teachers’ teaching performance, making them more inclined to choose conservative and low-risk teaching strategies (Muslimin et al., 2023). Teaching innovation usually requires additional attempts and tolerance for failure, which is more difficult to achieve under high pressure. Furthermore, in high techno-stress environments, teachers need to invest substantial time and energy in addressing technical problems, further limiting their capacity to implement innovative teaching strategies and technologies (Liu and Sun, 2026). For this reason, techno-stress is regarded as a key constraint for effectively integrating technology into teaching practice (Al-Adwan et al., 2024). In summary, excessive technological demands can reduce teachers’ commitment to and engagement in innovative teaching practices, causing them to gradually shift from being educators who actively explore pedagogical innovation to mere implementers who are overwhelmed by managing technological systems. To achieve deep integration of technology with teaching practice, reducing techno-stress among university teachers should serve as an important intervention entry point. Based on these arguments, this study proposes this hypothesis:

*H1*: Techno-stress has a significant negative correlation with teaching innovation.

### 1.3 The mediating role of job burnout

Job burnout refers to a comprehensive set of psychological symptoms, including emotional exhaustion, depersonalization, and reduced personal accomplishment, that individuals experience under prolonged work-related stress (Maslach and Jackson, 1981). From the perspective of JD-R Model (Demerouti et al., 2001), job burnout represents the depletion of psychological and emotional resources, a state that is theoretically counterproductive to the resource-demanding process of teaching innovation. Relevant studies have found that teachers’ techno-stress and job burnout have a significant positive correlation (Malaquias and de Souza, 2026). The cognitive burden imposed by techno-stress is accompanied by heightened anxiety and frustration. When teachers struggle to control the rhythm of their work, a steady decline into job burnout is a familiar outcome (Pagán-Garbín et al., 2024). At the same time, teachers must constantly switch between teaching, research, and technical tasks, while continuously dealing with highly unpredictable emergencies such as learning new software and technical glitches. The resulting technical complexity and sense of technical insecurity intensify the drain on their emotional and cognitive resources, ultimately manifesting as emotional exhaustion (Chen S. Z. et al., 2026). For example, the study by Asensio-Martínez et al. (2026) found that techno-stress prompts teachers to divert substantial time and energy from core teaching and research tasks to technical problem-solving, which not only weakens their engagement in primary duties but also threatens their physical and mental health. It can be seen from this that techno-stress is a key risk factor driving teachers’ job burnout.

Furthermore, a negative correlation exists between job burnout and teaching innovation (Caracuel Cáliz et al., 2025). This means that under conditions of severe job burnout, teachers show less enthusiasm for and reduced ability to try out novel instructional approaches (Monreal-Torres et al., 2025). For example, a study by Dahabiyeh et al. (2022) found that teachers experiencing higher levels of online teaching fatigue were less productive in areas such as course design, student feedback, and teaching innovation. Teachers often face varying degrees of stress, burnout, and work difficulties in the process of learning new technologies and preparing course content (Nashed et al., 2022). The daily demands of teaching consume substantial resources, leaving teachers with limited energy to invest in pedagogical exploration. Under these conditions, student-centered teaching methods are less likely to be implemented effectively in actual classroom settings (Romero et al., 2023). Therefore, an in-depth exploration of the relationship between job burnout, techno-stress, and teaching innovation can not only theoretically reveal the underlying psychological mechanisms by which techno-stress inhibits teaching innovation, but also provide empirical evidence for alleviating the technological burden on university teachers and stimulating teaching innovation. Based on the above, this study proposes the subsequent hypotheses:

*H2*: Techno-stress has a significant positive correlation with job burnout.

*H3*: Job burnout has a significant negative correlation with teaching innovation.

*H4*: Job burnout mediates the relationship between techno-stress and teaching innovation.

### 1.4 The mediating role of AI self-efficacy

AISE refers to an individual’s belief and confidence in the knowledge, skills, and coping abilities required to complete specific tasks using AI technology (Demirci et al., 2026). Existing studies have shown that techno-stress has a significant negative correlation with individual self-efficacy (Hu et al., 2025). For example, Liu and Sun (2026) found that the overload and uncertainty associated with techno-stress deplete teachers’ time and cognitive resources, increase their psychological burden, and undermine their sense of control over tasks. Lower self-efficacy tends to appear alongside these experiences. Furthermore, teachers who lack formal training and fear being overtaken by their students’ digital skills frequently experience cognitive overload. That pressure can reinforce learned helplessness and a fixed belief that they cannot master AI, both of which are associated with reduced AISE (Wu et al., 2026). Therefore, by systematically reducing techno-stress, it can help teachers build a more stable and resilient sense of AISE.

At the same time, AISE has a significant positive correlation with teaching innovation (Al-Mahdy and Elwakil, 2026). For instance, the research by Hastomo et al. (2025) reported that higher AISE among teachers correlates with viewing AI as a controllable teaching tool, and thus dare to break through the traditional teaching paradigm for innovative practice. This confidence is associated with a reduction in teachers’ psychological burden regarding potential operational failures, as well as with increased initiative and perseverance when facing technical challenges, alongside decreased anxiety about innovation (Gribiea, 2026). Additionally, high AISE is often accompanied by practical operational competence. AI-based teaching skills training enables teachers to proficiently use technology to complete tasks such as grading and information retrieval (Lu et al., 2024). Greater proficiency in this regard is accompanied by higher self-efficacy and increased engagement in innovative teaching (Li et al., 2025). In contrast, techno-stress is accompanied by a reduced sense of self-efficacy and an increase in psychological resistance to innovation (Zhang, 2023). Technical self-efficacy can effectively mitigate the obstacles that techno-stress poses to teaching innovation (Shan and Rasool, 2025). In summary, this study deeply examines the relationships among techno-stress, AISE, and teaching innovation, aiming to reveal the key value of AISE in mitigating the inhibitory effect of techno-stress on teaching innovation and stimulating innovative behaviors among university teachers. Based on the above, this study proposes the subsequent hypotheses:

*H5*: Techno-stress has a significant negative correlation with AISE.

*H6*: AISE has a significant positive correlation with teaching innovation.

*H7*: AISE mediates the relationship between techno-stress and teaching innovation.

### 1.5 The chain mediating role of job burnout and AI self-efficacy

Studies have found a significant negative correlation between teacher job burnout and self-efficacy (Angelini et al., 2026). From the perspective of Conservation of Resources (COR) theory (Hobfoll, 1989), job burnout represents a state of sustained depletion of an individual’s psychological resources, whereas self-efficacy is a domain-specific cognitive belief formed under resource constraints (Bandura, 1997). Resource depletion and individuals’ confidence appraisals toward new domains (e.g., AI technologies) are inherently intertwined under such constrained conditions. When teachers are in a state of high job burnout, their attention and energy are mainly used to complete daily teaching tasks and deal with psychological pressure, making it difficult for them to invest sufficient cognitive and emotional resources to learn new technologies, thus showing a relatively low sense of self-efficacy (Weissenfels et al., 2022). COR theory further posits that in times of resource scarcity, individuals tend to direct their remaining resources toward preventing further resource loss rather than pursuing new resource gains (Hobfoll, 2001). Crucially, this resource allocation pattern is asymmetrical: resource loss spirals operate more rapidly than gain spirals, making the defense of existing resources a temporal priority over the exploration of new ones. Moreover, the formation of AISE, as a new cognitive belief system, requires active cognitive engagement, trial, and error processing, all of which demand substantial cognitive and emotional resources that are precisely what burnt-out teachers lack. Thus, while low AISE might theoretically increase anxiety, it does not directly deplete the fundamental psychological resources in the same way that burnout does. This asymmetry in resource dynamics provides a theoretical basis for positing burnout as a temporal antecedent to, rather than merely a correlate of, AISE. This resource investment prioritization explains, from a temporal and allocational standpoint, why burnout emerges prior to and subsequently constrains the formation of AISE. Taking the adoption of AI as an example, new technologies bring techno-stress often accompanied by job burnout (Kim and Lee, 2024). Meanwhile, teachers’ sense of happiness is closely related to their self-efficacy in AI learning (Gribiea, 2026). When teachers lack confidence in their own AI skills, their willingness to try new technologies and drive teaching innovation is also limited (Al-Mahdy and Elwakil, 2026). In sum, job burnout, as a resource-depletion state, is associated with lower AISE among teachers. These two factors jointly constitute a sequential chain mediating pathway between techno-stress and teaching innovation. Therefore, exploring the roles of job burnout and AISE in the relationship between techno-stress and teaching innovation helps to understand this relationship from the perspective of individual internal resources and provides a theoretical basis for developing targeted intervention strategies. Based on the above, this study proposes the subsequent hypotheses:

*H8*: Job burnout has a significant negative correlation with AISE.

*H9*: Job burnout and AISE play a chain mediating role between techno-stress and teaching innovation.

**Figure 1:**
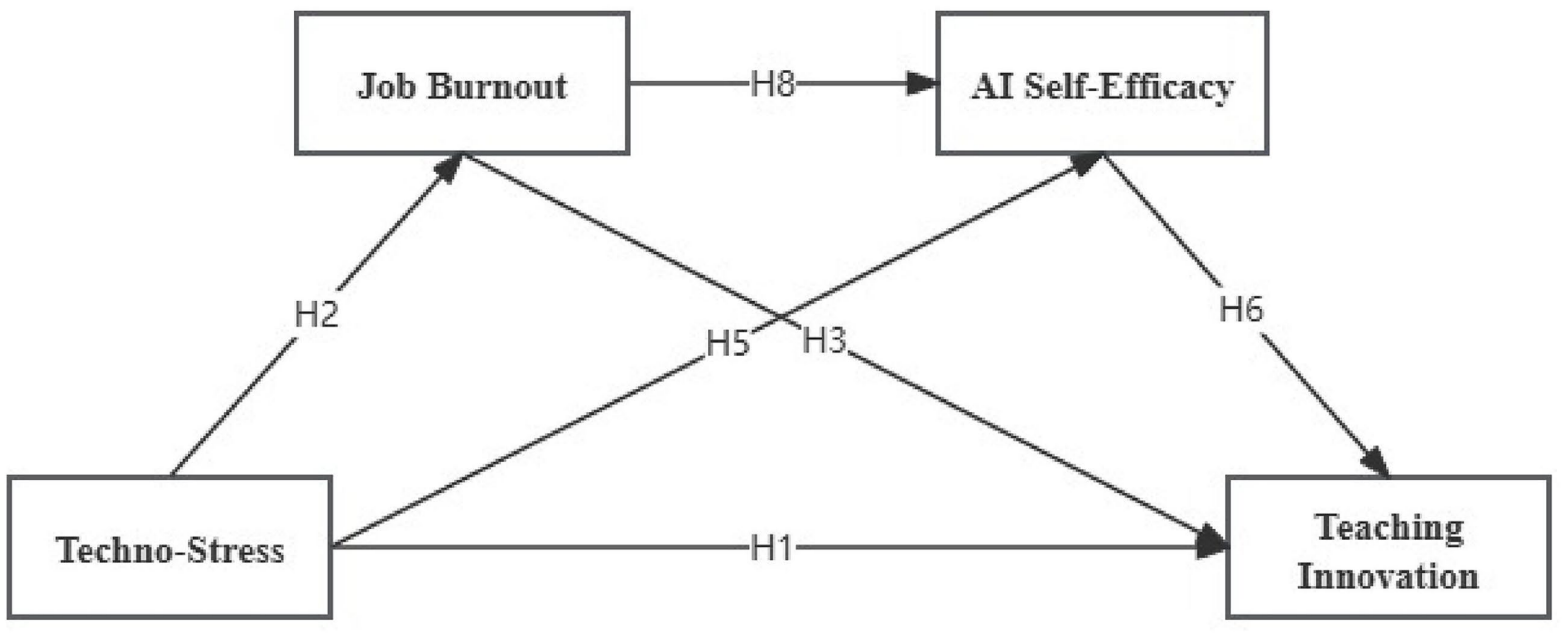
Hypothesis model.

Accordingly, this study proposes the theoretical framework shown in Figure 1.

## 2 Methods

### 2.1 Participants and data collection

This study conducted online data collection using a digital survey platform^1^ from February to March in 2026. Critically, this was not an open-access web survey; rather, it was a tightly administered, organization-assisted survey. To ensure the sample adequately represented the target population and to minimize selection bias, we employed a stratified random sampling strategy. The specific implementation process was as follows: Leveraging a higher education collaboration platform, we intentionally recruited 15 universities across 10 provinces (e.g., Beibu Gulf University), covering diverse institution types: 5 were comprehensive research universities, 6 were teaching-oriented undergraduate institutions, and 4 were vocational colleges; this set comprised 9 public and 6 private institutions. These universities were stratified into three geographical regions: eastern (*n* = 5), central (*n* = 4), and western (*n* = 6). According to the total number of eligible in-service teachers provided by each institution, we determined the proportional sample size for each stratum. The target sample fractions were 42% (eastern), 31% (central), and 27% (western), mirroring the national distribution of higher education faculty. After obtaining institutional permission, each participating university provided a complete roster of eligible in-service teachers. From these rosters, teachers were sequentially numbered, and a computer-generated random number table was used to draw numbers corresponding to the proportional sample size required for each institution, ensuring equal selection probability across all eligible participants. These selected teachers were then officially invited as survey participants.

Regarding the survey distribution and return process, it is important to clarify our operational definitions. The term “distributed” (823 copies) refers to the total number of unique survey links that were generated and sent via official institutional channels. Specifically, through each participating university’s human resources or academic affairs office, which distributed the links to the selected teachers’ institutional email addresses or enterprise WeChat accounts. The term “returned” (823 copies) is defined as the number of unique respondents who completed the entire questionnaire by the predetermined deadline. We achieved a 100% return rate not because the survey was self-selected or publicly accessible, but because each participating university appointed a dedicated liaison who actively monitored and followed up on completion progress. This administrative coordination, embedded within the formal university system, ensured full participation from all invited teachers. Consequently, the 100% return rate should be understood as a reflection of the effectiveness of these institutional organizational efforts, rather than as a natural response rate typical of open online surveys. All participants were fully informed of the study’s objectives, content, privacy protection measures, and the principle of voluntary participation. Only after voluntarily signing the written informed consent form can the formal investigation stage be initiated. In practice, all invited teachers consented to participate, and none withdrew during the process. This study was conducted in accordance with the ethical standards of the Institutional Review Board of Beibu Gulf University, which approved the research protocol (Approved No. BBGU-SPE-02). The entire research process strictly followed ethical guidelines, and all procedures complied with human research guidelines.

Following the sample size estimation method proposed by Kline (2023), each item required at least 10 respondents. This research questionnaire consists of a total of 61 items: 56 scale items across the four instruments (23, 22, 6, and 5 items, respectively) plus 5 demographic items. Based on an estimated 20% invalid questionnaire or sample loss rate, the minimum sample size required is calculated to be 732 people. The final number of invited participants (823) exceeded this minimum requirement. Prior to analysis, we conducted a systematic data screening process using the following criteria: (1) A validity threshold was established at an 80% item completion rate, whereas questionnaires with a missing data rate surpassing 20% were excluded from the analytic sample. For residual missing entries not meeting the deletion standard, missing values were addressed through the application of multiple imputation procedures. (2) Exclude questionnaires where responses show a clear tendency toward extreme reactions, such as those where all answers are concentrated on extreme options like “Strongly disagree” or “Strongly agree.” (3) Following the methodological framework proposed by Meade and Craig (2012), an absolute cutoff was established at the 3rd percentile of the overall response time distribution, corresponding to 100 s. Any participant whose total completion time dropped below this designated threshold was subsequently removed from the analytic sample. (4) As a unique quality control measure, our platform automatically assigns a unique tracking code to each participant’s invite link. This confirmed that all 823 responses originated from the intended, pre-selected participants with no duplicate or external submissions. Importantly, this tracking mechanism was designed solely for access control and duplication prevention; the code was cryptographically hashed upon data submission and stored separately from the survey responses, ensuring that the analytical dataset remained fully anonymized while preserving data integrity. After applying these criteria, 4 cases were excluded (1 for excessive missing data, 2 for extreme response straight-lining, and 1 for completion time below the 100-s threshold), leaving 819 valid responses for the final analysis (usable return rate of 99.51%). Participant demographic details are summarized; see Table 1. The sample is well-distributed across key variables, indicating that it is representative and capable of meeting the analytical requirements of subsequent research.

**Table 1:** Distribution of demographic characteristics.

Demographic characteristic variables	Category	Amount	Percentage (%)
Gender	Male	441	53.85
	Female	378	46.15
Age	Under 30 years old	134	16.36
	31–40 years old	268	32.72
	41–50 years old	230	28.09
	51 years old and above	187	22.83
Educational background	Undergraduate degree	144	17.58
	Master’s degree	253	30.89
	Doctor	422	51.53
Work experience	0–3 years	169	20.63
	4–7 years	231	28.21
	8–15 years	251	30.65
	16 years or more	168	20.51
Job level	Lecturer	279	34.07
	Associate professor	317	38.70
	Professor	223	27.23

### 2.2 Measurement instruments

#### 2.2.1 Techno-stress

This study used the scale developed by Tarafdar et al. (2014) to assess teachers’ techno-stress. This scale consists of 23 items, covering five dimensions: technical overload, technical intrusion, technical complexity, technical insecurity, and technical uncertainty. A sample item is “I have to sacrifice holidays or rest time to master new information technology tools.” The scale uses a 5-point rating system, with higher scores indicating greater techno-stress. In the Chinese sample, this scale has been validated as having good reliability and validity (Yang et al., 2025). In this study, the second-order confirmatory factor analysis (CFA) results indicated favorable construct validity (χ^2^/df = 1.861, RMR = 0.022, IFI = 0.984, RFI = 0.958, GFI = 0.959, CFI = 0.984, NFI = 0.966, TLI = 0.980, RMSEA = 0.032).

#### 2.2.2 Job burnout

This study used the Job Burnout Scale developed by Maslach and Jackson (1981) to assess teachers’ job burnout. The scale consists of 22 items across three dimensions: emotional exhaustion, depersonalization, and reduced personal accomplishment. A sample item is “Dealing with students and colleagues all day makes me feel a lot of stress.” A 5-point scale is employed for scoring; greater numerical values indicate increased burnout levels. This scale has demonstrated good reliability and validity in Chinese samples (Yaqian et al., 2024). In this study, the second-order CFA results indicated favorable construct validity (χ^2^/df = 2.354, RMR = 0.026, IFI = 0.974, RFI = 0.947, GFI = 0.944, CFI = 0.974, NFI = 0.956, TLI = 0.969, RMSEA = 0.041).

#### 2.2.3 AI self-efficacy

To assess teachers’ AISE, this study employed a scale originally developed by Laver et al. (2012) to measure computer self-efficacy, and subsequently adapted and validated for the AI context by Ding et al. (2025). The adaptation process involved a systematic substitution of the core technology term in each item, replacing all references to “computer” with “AI tools” while carefully preserving the original sentence structure, response format, and conceptual essence to ensure that the adapted scale maintained semantic equivalence with the original measure. The adapted items were reviewed by three experts in educational technology and pilot-tested with 30 university teachers to ensure clarity and contextual relevance. Minor wording adjustments were made based on their feedback. The scale contains six items, such as “When facing technical difficulties with AI use, I trust my ability to solve them.” Participants answered using a 5-point Likert scale, meaning that larger numbers signify greater AISE. In their validation study, Ding et al. (2025) administered the adapted scale to a sample of university teachers in China and reported excellent internal consistency reliability for the instrument, with a Cronbach’s *α* coefficient of 0.92. Further validation of the adapted scale in our own sample showed ideal construct validity (χ^2^/df = 2.633, RMR = 0.021, IFI = 0.992, RFI = 0.975, GFI = 0.991, CFI = 0.992, NFI = 0.987, TLI = 0.985, RMSEA = 0.045).

#### 2.2.4 Teaching innovation

This study used the scale originally developed by Liu et al. (2016) and subsequently adapted by Cui and Yin (2023) to assess teachers’ innovation in teaching. The scale consists of five items, such as “I actively explore and experiment with new teaching approaches to enhance students’ cognitive engagement.” A 7-point scoring system is adopted, and the higher the score, the higher the level of teaching innovation. This scale has demonstrated good reliability and validity in Chinese samples (Zhao and Huang, 2025). In this study, the scale showed ideal construct validity (χ^2^/df = 2.623, RMR = 0.036, IFI = 0.995, RFI = 0.985, GFI = 0.993, CFI = 0.995, NFI = 0.992, TLI = 0.991, RMSEA = 0.045).

### 2.3 Statistical analysis

Data were processed using SPSS 27.0 in conjunction with AMOS 28.0. The analytic workflow began with descriptive analyses, then progressed to reliability and validity evaluations of the study scales. Thereafter, CFA was conducted to ascertain measurement model fit quality and to investigate whether common method bias (CMB) posed a concern. Subsequently, group difference analyses were conducted. Finally, the covariance structural equation model (CB-SEM) was applied to test the significance of the structural model and path assumption and conduct the mediating effect analysis. Techno-stress and job burnout were specified as second-order latent constructs in AMOS, with their respective subdimensions serving as first-order indicators and the scale items serving as indicators of each subdimension. Given the potential biases in mediation analyses based on cross-sectional data noted in the methodological literature (Maxwell and Cole, 2007), this study did not employ chain mediation testing procedures based on ordinary least squares regression (e.g., the PROCESS macro). Because this method is difficult to effectively distinguish the temporal relationships among variables when dealing with cross-sectional data, it may lead to systematic biases in parameter estimation. Therefore, CB-SEM was selected as the core analytical tool based on the following considerations. First, this study is theory-testing in nature, aiming to evaluate the plausibility of a theoretically derived chain mediation model based on the JD-R framework. CB-SEM is the preferred approach for confirmatory theory evaluation, as it provides global fit indices such as CFI and RMSEA that assess the overall consistency between the hypothesized model and the observed data. This feature is not available in variance-based SEM (VB-SEM), which is more suited for prediction-oriented research (Hair et al., 2011; Hair et al., 2019b). Second, CB-SEM simultaneously estimates measurement and structural parameters, thereby accounting for measurement errors in both independent and mediating variables. This capability is especially critical for complex mediation models (Cole and Maxwell, 2003). Third, the sample size (*N* = 819) comfortably supports CB-SEM estimation, with a ratio of observations per parameter that well exceeds the recommended threshold of 5–10 (Bentler and Chou, 1987). For these reasons, VB-SEM was deemed less appropriate for the present confirmatory research context. With the objective of additional curtailment of estimation bias in standard errors, the mediation model was estimated using a bias-corrected percentile bootstrapping approach with 5,000 replications. Owing to its independence from strict distributional assumptions regarding normality, this strategy produces more dependable interval estimates for indirect effects (Preacher and Hayes, 2008).

## 3 Results

### 3.1 Descriptive statistics and correlation test

To ensure that the data met the assumptions of multivariate analysis, this study examined data normality by testing skewness and kurtosis, and used mean (M) and standard deviation (SD) for descriptive statistical analysis of variables that followed a normal distribution. According to Kim (2013), data are considered to be approximately normally distributed when the absolute skewness is less than 2 and the absolute kurtosis is less than 7. As shown in Table 2, all the variables in this study meet the requirements of a normal distribution.

**Table 2:** Correlation analysis and variable description.

Variables	M ± SD	Skewness	Kurtosis	Techno-stress	Job burnout	AISE	Teaching innovation
Techno-stress	2.802 ± 0.646	0.089	−0.769	1			
Job burnout	2.874 ± 0.661	−0.015	−0.767	0.565***	1		
AISE	2.930 ± 0.783	−0.005	−0.854	−0.501***	−0.528***	1	
Teaching innovation	3.889 ± 1.267	0.037	−0.783	−0.431***	−0.462***	0.454***	1

*N* = 819, path significance: ***p < 0.001; AISE, AI Self-Efficacy; the same below.

Correlation analysis shows that techno-stress has a significant positive correlation with job burnout, and a significant negative correlation with AISE and teaching innovation. Job burnout is significantly negatively correlated with AISE and teaching innovation. There is a significant positive correlation between AISE and teaching innovation. Overall, the direction of relationships among variables was consistent with the predictions derived from theoretical reasoning.

### 3.2 Reliability and validity testing

A rigorous and systematic evaluation of scale reliability and validity was undertaken in this study to ensure a complete verification of the measurement tools’ stability and overall soundness. We first examined the psychometric properties of techno-stress and job burnout at the dimensional level. As shown in Table 3, for techno-stress, the standardized factor loadings of its 23 items across the five dimensions ranged from 0.760 to 0.843, all exceeding the 0.50 benchmark (*p* < 0.001). The Cronbach’s alphas for these dimensions ranged from 0.836 to 0.869, composite reliabilities (CR) from 0.884 to 0.905, and average variances extracted (AVE) from 0.604 to 0.682. Similarly, for job burnout, the 22 items loading on the three dimensions yielded factor loadings between 0.712 and 0.782. The Cronbach’s alphas ranged from 0.822 to 0.897, CR from 0.876 to 0.916, and AVE from 0.545 to 0.585. For the single-dimensional measures, the AISE scale demonstrated factor loadings ranging from 0.711 to 0.768, with a Cronbach’s alpha of 0.839, CR of 0.882, and AVE of 0.554; the teaching innovation scale showed factor loadings from 0.754 to 0.859, with a Cronbach’s alpha of 0.860, CR of 0.899, and AVE of 0.641. All reliability coefficients exceeded the 0.70 criterion, and all AVE values surpassed the 0.50 threshold, confirming satisfactory internal consistency and convergent validity for all lower-order dimensions (Hair et al., 2021; Henseler et al., 2015).

**Table 3:** Reliability and validity.

Variables	Item	Loading		Cronbach’s alpha	CR	AVE
Techno-stress	TO1	0.830	0.890	0.869	0.905	0.656
	TO2	0.817				
	TO3	0.825				
	TO4	0.781				
	TO5	0.796				
	TIT1	0.843	0.886	0.844	0.896	0.682
	TIT2	0.827				
	TIT3	0.820				
	TIT4	0.812				
	TC1	0.760	0.918	0.836	0.884	0.604
	TC2	0.784				
	TC3	0.780				
	TC4	0.768				
	TC5	0.792				
	TIS1	0.793	0.907	0.847	0.891	0.620
	TIS2	0.777				
	TIS3	0.787				
	TIS4	0.791				
	TIS5	0.789				
	TU1	0.805	0.861	0.836	0.891	0.671
	TU2	0.800				
	TU3	0.840				
	TU4	0.831				
Job burnout	EE1	0.747	0.969	0.897	0.916	0.549
	EE2	0.754				
	EE3	0.747				
	EE4	0.753				
	EE5	0.740				
	EE6	0.740				
	EE7	0.720				
	EE8	0.723				
	EE9	0.736				
	DP1	0.750	0.936	0.822	0.876	0.585
	DP2	0.781				
	DP3	0.782				
	DP4	0.760				
	DP5	0.749				
	PA1	0.712	0.963	0.881	0.905	0.545
	PA2	0.747				
	PA3	0.725				
	PA4	0.734				
	PA5	0.747				
	PA6	0.749				
	PA7	0.751				
	PA8	0.739				
AISE	AISE1	0.714		0.839	0.882	0.554
	AISE2	0.761				
	AISE3	0.759				
	AISE4	0.768				
	AISE5	0.751				
	AISE6	0.711				
Teaching innovation	TI1	0.859		0.860	0.899	0.641
	TI2	0.797				
	TI3	0.791				
	TI4	0.800				
	TI5	0.754				

TO, technical overload; TIT, technical intrusion; TC, technical complexity; TIS, technical insecurity; TU, technical uncertainty; EE, emotional exhaustion; DP, depersonalization; PA, reduced personal accomplishment; AISE, AI self-efficacy; TI, teaching innovation. The same below.

However, the inter-construct correlations among the five techno-stress dimensions were relatively high, and similarly, the inter-construct correlations among the three job burnout dimensions were also high, suggesting limited discriminant validity at the dimensional level in our sample. This pattern implies that, within the specific Chinese educational context, teachers may perceive techno-stress and job burnout as holistic, integrated experiences rather than as empirically distinct multifaceted constructs. From a theoretical standpoint, such dimensional coalescence is consistent with the “holistic” or “gestalt” perspective commonly observed in collectivist cultural settings, where individuals tend to process psychological states in a more global and integrated manner, rather than through fine-grained dimensional differentiation (Markus and Kitayama, 1991; Triandis, 2018). Moreover, when lower-order dimensions exhibit extremely high intercorrelations, they may reflect a common overarching latent syndrome, thereby justifying their aggregation into a unidimensional composite. As Gerbing and Anderson (1988) argued, if the correlations among subdimensions approach unity, treating them as separate factors introduces unnecessary model complexity without improving explanatory power; in such cases, a higher-order or unidimensional representation is statistically and theoretically defensible. To formally evaluate the viability of this higher-order representation, we conducted second-order CFA for measurement model, it yielded excellent fit indices: χ^2^/df = 1.413, SRMR = 0.034, IFI = 0.977, RFI = 0.919, GFI = 0.914, CFI = 0.977, NFI = 0.925, TLI = 0.975, RMSEA = 0.022. These results confirm that a single higher-order factor adequately accounts for the covariation among the sub-dimensions. The standardized factor loadings of the lower-order dimensions on their respective higher-order constructs are also presented in Table 3. Methodologically, treating these constructs as unidimensional not only preserves parsimony but also alleviates concerns about multicollinearity, which can inflate standard errors and destabilize parameter estimates in regression-based models (Hair et al., 2019a). Given that our primary interest lies in the global evaluative effects of techno-stress on overall job burnout, rather than in the unique contributions of each subdimension, aggregating to the construct level aligns with both our theoretical focus and empirical evidence. Given the well-fitting second-order CFA results, which confirmed that a single higher-order factor adequately accounts for the covariation among the sub-dimensions, we operationalized both techno-stress and job burnout as unidimensional composite constructs for subsequent hypothesis testing.

As shown in Table 4, the heterotrait-monotrait (HTMT) ratios among all latent variables were well below the stringent threshold of 0.85. Meanwhile, as shown in Table 5, the square roots of each latent variable AVE are significantly greater than their correlation coefficients with other variables. Taken together, these results confirm that the scale has good discriminant validity (Hair et al., 2021). Notably, The high Cronbach’s alpha values (job burnout: 0.954; techno-stress: 0.957) may suggest some item redundancy, particularly as both AVEs are just above 0.50. However, this concern is mitigated by two observations. First, all HTMT ratios range from 0.473 to 0.592 (Table 4), well below the 0.85 threshold, confirming discriminant validity. Second, inter-construct correlations are moderate (|*r*| = 0.431–0.565) (Table 2), ruling out a single underlying construct. Thus, while item overlap may exist within each scale, the constructs remain empirically distinct.

**Table 4:** The HTMT.

Variables	Techno-stress	Job burnout	AISE	Teaching innovation
Techno-stress				
Job burnout	0.592			
AISE	0.560	0.589		
Teaching innovation	0.473	0.506	0.534	

**Table 5:** The Fornell-Larcker criterion.

Variables	Techno-stress	Job burnout	AISE	Teaching innovation
Techno-stress	***0.717***			
Job burnout	0.573	***0.715***		
AISE	−0.506	−0.530	***0.744***	
Teaching innovation	−0.433	−0.462	0.455	***0.801***

The diagonal entries of the table display the square roots of AVE values, formatted in bold and italic.

### 3.3 Common method bias

The data used in this study were all self-reported by the subjects and there may be CMB. To address this, a preliminary analysis using Harman’s single-factor method was first undertaken in the study. The results of exploratory factor analysis showed that six factors had eigenvalues greater than 1, with the first factor explaining 36.298% of the variance, which did not exceed the 40% threshold (Podsakoff et al., 2003), preliminarily indicating that CMB was not a serious concern. Given that the Harman’s single-factor test has limitations such as overly strong assumptions and low sensitivity, this study adopts the more rigorous unmeasured latent method construct as the primary testing approach, and uses the Harman test only as a supplementary reference (Podsakoff et al., 2012). CFA was initially performed on the measurement model to validate the constructs under investigation, and this initial structure was adopted as the reference point. Following this step, an additional latent factor representing common method variance was incorporated into the baseline model, with all item loadings on this factor subjected to equal parameter constraints, thus generating a competing control model. Through a comparative evaluation of the fit indices generated by both models, it was possible to detect whether there was a significant CMB.

Given the ordinal categorical nature of the responses, robust estimation methods, such as the weighted least squares mean and variance adjusted approach (WLSMV) or maximum likelihood estimation with robust standard errors (MLR) (Brauer et al., 2023; Li, 2016). Since AMOS was the analytic tool employed herein and its default setting invokes maximum likelihood method (ML) estimation, this approach was naturally followed. The application of ML is defensible in our specific context based on three considerations. First, diagnostic tests for normality (reported in Table 2) confirmed that all univariate skewness and kurtosis estimates were within the acceptable ranges, satisfying the continuous-data assumption at the item level. Second, given that our Likert-type items feature more than four response categories, prior methodological research has demonstrated that ML estimation performs well, producing unbiased coefficients and accurate standard errors under such conditions, even with ordinal data (Finney and DiStefano, 2006; Rhemtulla et al., 2012). Third, and most importantly, we addressed the remaining concern regarding multivariate normality by employing bootstrapping techniques (with 5,000 resamples) to compute robust standard errors and bias-corrected percentile confidence intervals. This nonparametric adjustment effectively mitigates the over-rejection of the chi-square test and compensates for the absence of the MLR option in AMOS (Nevitt and Hancock, 2001). Under such circumstances, the application of ML estimation continues to generate parameter estimates that are reasonably robust, notwithstanding the ordinal categorical measurement scale of the data (Kline, 2023). Accordingly, our estimation strategy balances methodological rigor with practical software constraints.

Table 6 reveals that acceptable overall fit indices were obtained for both two models (Hu and Bentler, 1998). Differential evaluation criteria were sourced from the invariance-testing literature (Chen, 2007; Cheung and Rensvold, 2002), with every empirical discrepancy registering well below the recommended values. This pattern of results points to the conclusion that augmenting the model with a common method factor did not produce a practically significant gain in fit. Moreover, after controlling for the method factor, all 56 indicators retained statistically significant loadings on their intended trait factors (all *p* < 0.001), well above the 0.50 threshold (Hair et al., 2019a). Compared with the baseline model, the decreases in factor loadings ranged from −0.007 to −0.062, with a maximum drop of only 0.062, substantially below the commonly used 0.10 concern threshold (Podsakoff et al., 2003). The variances of the four constructs decreased by 10.7, 12.3, 11.1, and 1.5%, respectively, all well below the 30% benchmark (Williams et al., 1989). CMB is not significant in the measurement model of this study, supplying a more robust statistical underpinning for the conclusion derived from the foregoing Harman single-factor analytic approach.

**Table 6:** Model comparison results.

Index	*χ* ^2^ */df*	RMSEA	SRMR	GFI	NFI	CFI	IFI	TLI	RFI
Reference value	<3	<0.06	<0.06	>0.9	>0.9	>0.9	>0.9	>0.9	>0.9
Baseline model	1.413	0.022	0.034	0.914	0.925	0.977	0.977	0.975	0.919
Control model	1.410	0.022	0.034	0.915	0.925	0.977	0.977	0.975	0.919
Difference	0.003	<0.001	<0.001	0.001	<0.001	<0.001	<0.001	<0.001	<0.001
Judgment standard		Δ < 0.01	Δ < 0.025	Δ < 0.01	Δ < 0.01	Δ < 0.01	Δ < 0.01	Δ < 0.02	Δ < 0.01

### 3.4 Collinearity test

To ensure the robustness of the model and the reliability of the analysis conclusions, this study uses the variance inflation factor (VIF) to diagnose and evaluate the multicollinearity problem among the respective variables. The results presented in Table 7 showed that the VIF values of all predictor variables were below 3.3, indicating that multicollinearity was not a concern (Hair et al., 2019c).

**Table 7:** The results of collinearity test.

Variables	VIF
Techno-stress	1.604
Job burnout	1.664
AISE	1.513

### 3.5 Analysis of group differences

To examine the differences in teaching innovation across various demographic characteristics, this study conducted independent samples t-tests and one-way analysis of variance on gender, age, educational background, work experience, and job level. The results (Table 8) showed that teaching innovation exhibited between-group differences in gender and age.

**Table 8:** Test results.

Outcome variable	Covariate		M ± SD	t/F	*p*
Teaching innovation	Gender	Male (*n* = 441)	3.212 ± 1.037	20.197	<0.001
		Female (*n* = 378)	4.678 ± 1.033		
	Age	Under 30 years old (*n* = 134)	4.176 ± 1.208	5.687	0.001
		31–40 years old (*n* = 268)	3.812 ± 1.392		
		41–50 years old (*n* = 230)	4.008 ± 1.204		
		51 years old and above (*n* = 187)	3.646 ± 1.142		
	Educational background	Undergraduate degree (*n* = 144)	3.850 ± 1.369	1.134	0.322
		Master’s degree (*n* = 253)	3.806 ± 1.247		
		Doctor (*n* = 422)	3.952 ± 1.243		
	Work experience	0–3 years (*n* = 169)	3.787 ± 1.205	1.526	0.206
		4–7 years (*n* = 231)	3.796 ± 1.269		
		8–15 years (*n* = 251)	3.987 ± 1.273		
		16 years or more (*n* = 168)	3.971 ± 1.310		
	Job level	Lecturer (*n* = 279)	3.939 ± 1.302	1.773	0.170
		associate professor (*n* = 317)	3.940 ± 1.282		
		Professor (*n* = 223)	3.753 ± 1.196		

### 3.6 Structural model testing

**Figure 2:**
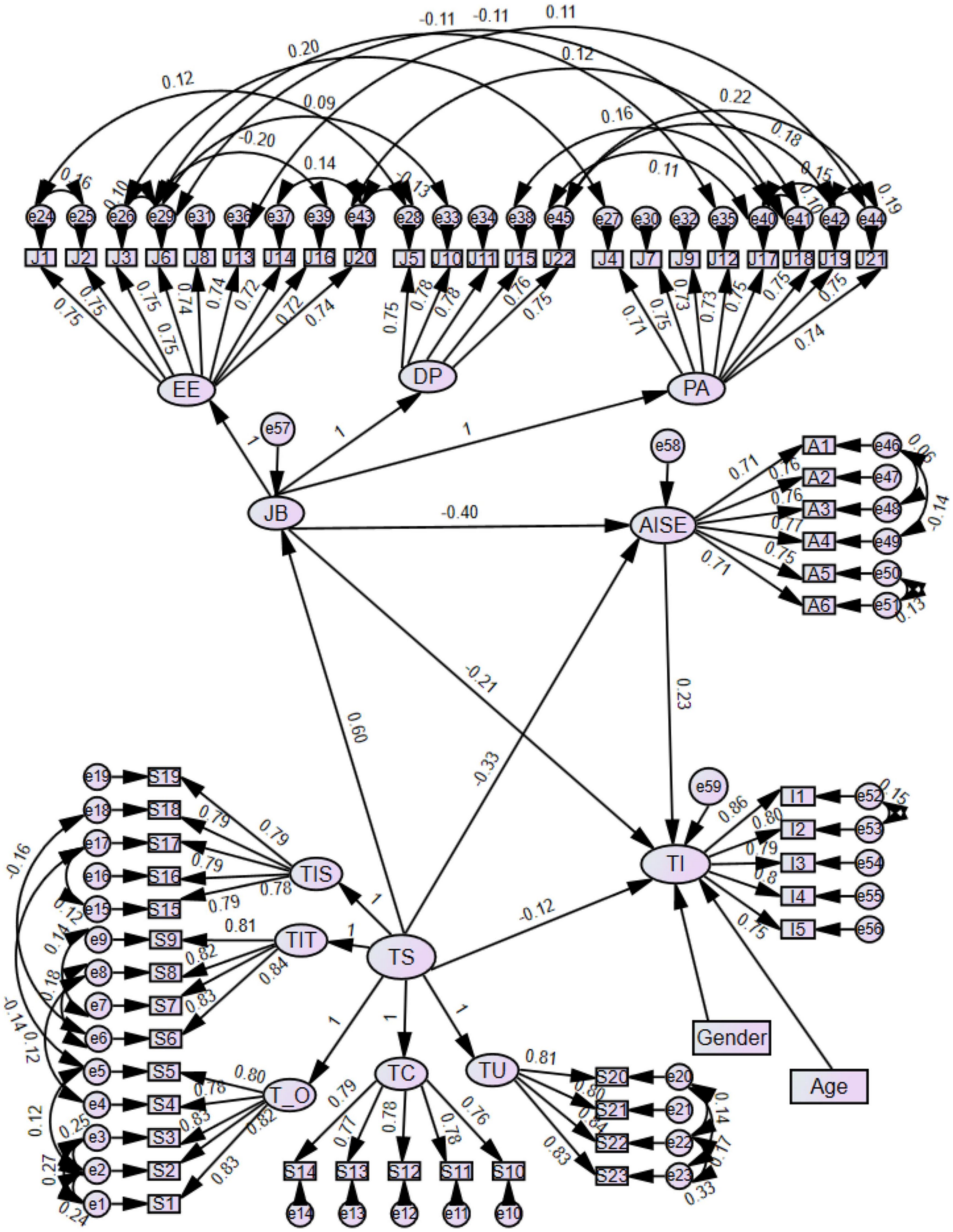
Structural model. TS, Techno-Stress; T_O, Technical Overload; JB, Job Burnout. *Gender* was dummy-coded (0 = male, 1 = female). *Age* was originally assessed using ordinal categories and was recoded to its category midpoint.

The structural model evaluation results showed that the model demonstrated ideal structural validity (χ^2^/df = 1.554, SRMR = 0.053, IFI = 0.967, GFI = 0.900, CFI = 0.967, NFI = 0.912, RFI = 0.906, TLI = 0.964, RMSEA = 0.026). To address the potential threat of reverse causality, we tested an alternative model reversing the overall hypothesized relationship (Y → M2 → M1 → X). This model yielded a substantially poorer fit (AIC = 3179.292, BIC = 3918.461) compared to our hypothesized model (AIC = 2729.166, BIC = 3473.043), with *Δ*AIC = 450.126 and ΔBIC = 445.418. Following the guidelines of Burnham and Anderson (2004) (Δ > 10 as decisive evidence against the inferior model), these results strongly rule out the reverse-causality interpretation. The subsequent path analysis (see Table 9 and Figure 2) revealed that H1, H2, H3, H5, H6, and H8 were all supported. To ensure the robustness of our findings, we included gender and age as control variables in the model; neither exhibited significant effects on teaching innovation (all *p* > 0.05), and their inclusion did not alter the significance or direction of the hypothesized paths. The R^2^ for teaching innovation was 0.504 [Bootstrap 95% CI = (0.448, 0.552), *p* = 0.002], indicating that the model explained 50.4% of its variance. The lower bound of this effect size (0.448) far exceeded the threshold of 0.26 that Cohen (1988) proposed for a large effect, demonstrating robust explanatory power. Multicollinearity diagnostics among the mediators showed that all VIF values were below 3.3 and all tolerance values exceeded 0.1, indicating no serious collinearity concerns.

**Table 9:** The test results of the path hypothesis.

Hypothetical path	*B*	β	SE	*T*	*p*	Results
Techno-stress → teaching innovation	−0.280	−0.121	0.096	−2.913	0.004	Supported
Techno-stress → job burnout	0.601	0.596	0.044	13.762	<0.001	Supported
Techno-stress → AISE	−0.356	−0.330	0.048	−7.383	<0.001	Supported
Job burnout → AISE	−0.423	−0.396	0.050	−8.534	<0.001	Supported
Job burnout → teaching innovation	−0.482	−0.211	0.100	−4.824	<0.001	Supported
AISE → teaching innovation	0.484	0.226	0.100	4.847	<0.001	Supported

B, unstandardized regression coefficient; β, standardized regression coefficient. The same below.

### 3.7 Mediating effects analysis

We tested the significance of the mediation effects using CB-SEM. The results indicated that the relationship between techno-stress and teaching innovation remained significant, showing a negative correlation between the two variables. As shown in Table 10, after controlling for the mediating variables, the direct effect between the two becomes apparent. The confidence intervals for all three mediation paths exclude zero, indicating that job burnout and AISE each play a partial mediating role between techno-stress and teaching innovation, and that the chained mediation path formed by these two factors is also statistically significant. Therefore, H4, H7, and H9 were all supported.

**Table 10:** Test of mediating role.

Effect	Path	*B*	β	Percentage	95% CI	
					LLCI	ULCI
Total effect	Techno-stress → teaching innovation	−0.864	−0.375	100%	−0.456	−0.302
Direct effect	Techno-stress → teaching innovation	−0.280	−0.121	32.27%	−0.213	−0.030
Indirect effect	Techno-Stress → job burnout → teaching innovation	−0.290	−0.126	33.60%	−0.187	−0.069
	Techno-stress → AISE → teaching innovation	−0.172	−0.075	20.00%	−0.120	−0.041
	Techno-stress → job burnout → AISE → teaching innovation	−0.123	−0.053	14.13%	−0.087	−0.029

## 4 Discussion

The findings reveal a significant negative correlation between techno-stress and teaching innovation, aligning with the results reported by Hernández (2025). Compared with studies based on Western samples, the techno-stress on Chinese university teachers is associated with the institutional situation of passive implementation rather than active exploration, which is reflected in a tendency to meet formal requirements at the lowest cost rather than invest in genuine teaching innovation (Liu and Sun, 2026). When techno-stress manifest as performance evaluation pressures, teachers are more likely to perceive them not as supportive opportunities for growth but as punitive risks of accountability; this perception is correlated with a tendency to scale back innovative practices and revert to traditional teaching methods (Wu et al., 2022). This pattern suggests that within the institutional context of Chinese universities, the relationship between techno-stress and teaching innovation is not an accidental psychological phenomenon, but rather an outcome associated with the imbalance between job requirements and resources.

A significant positive relationship existed between techno-stress and job burnout, and job burnout had a significant negative correlation with teaching innovation in university teachers. The analysis of the mediating effect further revealed that job burnout was found to partially mediate the relationship between the two. Specifically, Niu et al. (2022) pointed out that sustained techno-stress, accompanied by the continuous depletion of teachers’ emotional and cognitive resources, represents an important coexisting factor in the context of job burnout. Furthermore, the study by Monreal-Torres et al. (2025) found that long-term job burnout was associated with lower psychological energy reserves and reduced cognitive participation among teachers. Their exploration and attempts of new teaching methods tend to be less frequent in this context. Without adequate psychological support mechanisms, burnout is more prevalent in this context, and this prevalence is associated with reduced enthusiasm for investing in the high-risk, long-term process of pedagogical innovation. These findings imply that reducing techno-stress and addressing job burnout systematically, for instance by reframing technical indicators as developmental guidance rather than compliance measures and by establishing error-tolerant cultures alongside psychological support mechanisms, may be associated with sustaining university teachers’ motivation for teaching innovation.

A significant negative relationship existed between techno-stress and AISE, and AISE had a significant positive correlation with teaching innovation in university teachers. The results of the mediating effect analysis show that AISE plays a partial mediating role between techno-stress and teaching innovation. This conclusion is consistent with the findings of Liu and Sun (2026): when teachers are faced with excessive technological demands, systemic complexity, and uncertainties about future outcomes, their creative self-efficacy tends to remain consistently low. Consequently, they encounter considerable practical obstacles in their efforts to implement innovative pedagogical approaches and technological tools in classroom settings. Specifically, in institutional contexts where both research productivity and teaching performance are subjected to intensive evaluation, techno-stress is closely associated with a notably greater decline in teachers’ AISE (Wu et al., 2026). In this context, teachers’ confidence in AI tools is of vital importance. When AI tools are used appropriately, teachers’ job satisfaction and self-efficacy are often observed at relatively high levels, and teaching innovation is more frequently manifested (Ding et al., 2026). This finding offers a reference for higher education institutions: the digital transformation of education may need to address both the relief of techno-stress and the improvement of teachers’ AISE.

This study verified the chain mediating role of job burnout and AISE in the relationship between techno-stress and teaching innovation among university teachers. The techno-stress Chinese teachers encounter often have a stronger administrative component, such as mandatory requirements for smart classrooms and online course development. High work pressures and a scarcity of job resources coexist, with widespread burnout being a prominent feature of this context (Tu et al., 2025). Teachers in a state of long-term burnout, finding it difficult to invest sufficient time and energy in learning and practicing AI tools. Repeated failures and inefficient practices are pervasive, and self-efficacy among these teachers is correspondingly low (Angelini et al., 2026). Teachers with lower AISE are more likely to overestimate operational challenges and doubt their coping capacity, a cognitive pattern that corresponds with reduced engagement in AI-based innovation (Al-Mahdy and Elwakil, 2026). Thus, under intense, administratively driven pressure to adopt technology, Chinese university teachers appear to be caught in a double bind of burnout and a loss of self-efficacy regarding AI, making it difficult for them to pursue AI-based teaching innovation.

Beyond mere mean comparisons, the demographic variations in teaching innovation observed in this study invite theoretical interpretation through the lens of coping mechanisms and professional identity formation. The superior performance of female teachers in communication, methodological application, and AI-related domains (Martínez et al., 2022; Villegas-José and Delgado-García, 2024) may reflect their heightened sensitivity to relational pedagogies, which may facilitate the perception of technological innovations as extensions of existing instructional rapport. In contrast, male teachers’ stronger preference for gamification and experiential learning (Soler and Aliaga-Aguza, 2025) likely reflects differential risk-taking dispositions in pedagogical experimentation, wherein male educators may gravitate toward structurally novel, game-based formats that allow for more distal departures from traditional lecturing. Regarding age, the data did not support a straightforward “younger teachers innovate more” gradient. The group means were non-monotonic (under–30 = 4.176, 31–40 = 3.812, 41–50 = 4.008, 51 + = 3.646). Post-hoc tests showed that the youngest group significantly outperformed the 31–40 and 51 + groups, whereas the 41–50 group scored significantly higher than the oldest group. This pattern challenges the simplistic “younger-is-better” narrative, suggesting instead that mid-career teachers occupy an optimal equilibrium zone, one where sufficient digital fluency to engage with emergent tools like ChatGPT coexists with rich pedagogical content knowledge, enabling technology integration that is meaningfully transformative rather than superficially performative. Meanwhile, although older teachers showed relatively lower frequencies in AI tool integration, they accumulated richer experiential advantages in communication skills and interdisciplinary integration (Martínez et al., 2022; Villegas-José and Delgado-García, 2024). It is worth noting that teaching experience itself does not directly determine the degree to which teachers adopt generative AI tools such as ChatGPT, indicating that the generational technological adaptation differences reflected by age may be more explanatory than simple accumulation of experience (Jiang et al., 2025). In addition, the subject area in which teachers specialize and their cultural background also play a significant role in the assessment of their ability to innovate in teaching (Martínez et al., 2022).

## 5 Implications and limitations

### 5.1 Theoretical implication

Based on the JD-R Model, this study systematically revealed the theoretical relationships among techno-stress, job burnout, AISE, and teaching innovation in university teachers. First, the research reveals how techno-stress, an external job requirement, is systematically associated with internal psychological resources such as job burnout and AISE, as well as teaching innovation behaviors. Second, it elucidates the intrinsic link between job burnout and AISE, as well as how these two factors are jointly associated with teaching innovation. Within this theoretical framework, this study constructed an explanatory model that integrates external job demands with internal personal resources, deepening the theoretical understanding of the motivational mechanisms underlying teaching innovation in university teachers. Furthermore, research has found that there may be significant differences in teaching innovation among different groups of teachers, and these differences are closely related to their age and gender. By focusing on demographic variables, this study provides empirical evidence for the future development of a more inclusive and context-sensitive theoretical framework for teaching innovation among university teachers. In summary, from the perspective of integrating job demands and personal resources, this study systematically explained the formation mechanisms of teaching innovation in university teachers and their group differences, providing theoretical support for advancing research on teacher development within the setting of digital change occurring in universities.

### 5.2 Practical implication

The findings of this study not only deepen the theoretical understanding of the relationship between techno-stress and teaching innovation among university teachers, but also provide clear pathways for practical intervention.

Firstly, encourage teachers to actively seek professional support by regularly participating in teaching development workshops with designated consultation hours, engaging in formalized peer-observation partnerships with experienced colleagues, and other means when they encounter AI techno-stress or job burnout in teaching practice. In the real teaching interaction, they can receive technical empowerment, experience sharing and continuous improvement of teaching effectiveness. At the same time, they are encouraged to strengthen their sense of professional identity through activities such as monthly teaching reflection journals, curriculum design sharing sessions, and annual teaching innovation showcases, thereby alleviating the career anxiety caused by technological disruption.

Second, faculty development services in higher education should go beyond traditional skills training by redesigning structured AI competency development programs to include “teaching challenge articulation” modules, through which teachers systematically sort out and express the confusions and gains in their teaching practice. This structured articulation process, helps them better understand and accept the teaching challenges brought about by technological changes. Services should avoid treating teachers merely as passive recipients of AI technologies; instead, they should help teachers recognize their professional capabilities and potential for growth through learning and collaboration, with specific emphasis on peer-coaching circles that meet biweekly and action-learning projects that connect directly to teachers’ real classrooms, thereby strengthening their intrinsic sense of AISE and confidence in teaching innovation.

Third, institutional policies must address both the psychological and administrative dimensions of techno-stress. Specific adjustments are recommended: department heads should allocate dedicated time for technology-sharing agendas in regular meetings; teaching evaluation mechanisms should be adjusted to recognize pedagogical experimentation alongside successful outcomes; administrative burdens should be reduced by streamlining redundant data-entry requirements and consolidating digital reporting channels; and peer recognition should be enhanced through colleague-nominated teaching innovation awards. These measures, taken together, can create a supportive environment where teachers truly experience a dynamic and creative career through continuous learning and personal growth.

### 5.3 Limitations and future directions

This study provides a preliminary exploration of the relationship between techno-stress and teaching innovation among university teachers in the intelligent era, but several limitations should be considered when generalizing its findings. Firstly, the main limitation of this study lies in its cross-sectional design. Although the model proposes directional hypotheses based on theory and path analysis tests the theoretical relationships among variables, cross-sectional data cannot confirm causal sequences or determine temporal order. Therefore, it lacks the capacity for causal inference. Future research should employ longitudinal designs, using follow-up surveys or intervention experiments, to effectively examine the causal relationships among variables and their dynamic processes over time. Secondly, the data mainly comes from self-reports of university teachers, and there may be deviations in their subjective perception and evaluation of teaching innovation. Future research could integrate multiple sources of information, such as peer reviews, student feedback, teaching portfolios, or course design documents, to enhance the objectivity and interpretive power of the results. Third, the sample of this study consisted entirely of Chinese university teachers working in the context of the intelligent era. However, considering that the Chinese education system has a high degree of policy-driven and collectivist cultural tendency in technological integration, which is significantly different from the Western education system, such discrepancies could restrict the generalizability of the findings. To determine the extent to which the current findings hold across varying cultural environments, future studies ought to draw upon participants with heterogeneous cultural heritage and engage in systematic cross-cultural comparisons, with the aim of delineating both the limiting conditions and the transferability of the evidence reported herein. Fourth, it should be acknowledged that our data were measured on an ordinal Likert scale, yet we employed ML estimation via AMOS, which treats indicators as continuous and does not offer robust MLR or WLSMV estimators. Although we confirmed approximate univariate normality, this approach does not fully account for the categorical nature of the items, potentially affecting chi-square statistics and standard errors. We therefore list this as a methodological limitation. Future research is encouraged to re-examine our model using software that supports WLSMV or MLR to confirm the robustness of our findings.

## 6 Conclusion

Based on the JD-R Model, this study systematically examined the relationships among techno-stress, job burnout, AISE, and teaching innovation in university teachers. The results indicate that there is a significant negative correlation between techno-stress and teaching innovation, with job burnout and AISE playing a partial mediating role, thereby forming a chained mediation pathway. There are significant differences in teaching innovation among university teacher groups of different genders and ages. The findings support the applicability of the JD-R Model in the context of intelligent education and provide theoretical support and practical reference for promoting professional development and teaching transformation among university teachers. It bears emphasizing that the current investigation is primarily oriented toward cognitive and affective phenomena situated within the individual, and does not comprehensively address the regulatory contribution of extra-organizational or contextual factors. Moving forward, scholarly efforts ought to amalgamate macroscopic organizational parameters with microscopic psychological operations, thereby advancing a more exhaustive theoretical architecture that spans multiple levels of analysis.

## Data Availability

The raw data supporting the conclusions of this article will be made available by the authors, without undue reservation.

## Footnotes

1
www.Sojump.com
